# A practical optimization method for enhancing drilling efficiency in panel furniture manufacture

**DOI:** 10.1371/journal.pone.0339912

**Published:** 2026-01-16

**Authors:** Guokun Wang, Xianqing Xiong, Sijie Fu, Mei Zhang, Lujie Yang

**Affiliations:** 1 College of Furnishings and Industrial Design, Nanjing Forestry University, Nanjing, China; 2 Center for Renewable Carbon, School of Natural Resources, University of Tennessee, Knoxville, Tennessee, United States of America; 3 Co-Innovation Center of Efficient Processing and Utilization of Forest Resources, Nanjing Forestry University, Nanjing, China; University of Lagos Faculty of Engineering, NIGERIA

## Abstract

Drilling is one of the most complex processes in the manufacturing of panel-type furniture and has a significant impact on both production efficiency and takt-time stability. With the growing demand for large-scale customized furniture, the complexity of drilling operations has increased substantially. Moreover, variations in equipment capabilities further hinder the automation and standardization of this process. In this technical note, we ad-dress three common challenges in the drilling process of panel furniture: the inability to process horizontal holes located on fixed reference edges, low slotting efficiency, and pro-longed idle time of drilling equipment. To resolve these issues, we propose a set of strategies based on flipping, slot extension, and hole-slot logic transformation. The proposed methods have been successfully implemented in a large-scale customized panel furniture production line. Practical results demonstrate their effectiveness in improving drilling efficiency. Specifically, for panel components previously limited by low slotting efficiency, the optimization was applicable to 42.30% of the parts. Additionally, for panels that previously suffered from long idle periods during processing, the drilling efficiency increased by 56.01%. These findings confirm the practical value and applicability of the proposed approach in industrial settings, particularly in enhancing operational consistency and promoting automation in mass-customized furniture manufacturing.

## Introduction

Panel-type customized furniture has become a mainstream product in the furniture industry, offering design flexibility to meet a wide range of customer needs [1]. The production process of such furniture typically consists of four sequential stages: cutting, edge banding, drilling, and packaging [2]. Among these, the drilling stage is considered the most complex and technically demanding [3].

Currently, several studies have investigated structural improvements related to drilling components in panel-type furniture. For example, Krzyżaniak examined the mechanical performance of concealed detachable joints [4], while Kuşkun et al. conducted experimental and numerical studies on expanding dowels [5]. Kasal et al focused on enhancing L-type corner joints using auxetic connectors [6]. Bedelean et al. made further contributions by combining ANN (Artificial Neural Networks) and RSM (Response Surface Methodology) to model and improve drilling quality in MDF(Medium Density Giberboard) and wood-based composite panels [7,8]. More recent studies have emphasized drilling mechanics, chip evacuation, and process stability in engineered wood materials. For instance, Szwajka et al. analyzed drilling-induced defects and mechanical performance in wood-based panels and demonstrated how process conditions directly affect joint reliability [9].

Despite these efforts, research directly targeting the optimization of the drilling process itself remains limited. Wang et al. briefly considered drilling parameters within a broader production scheduling context [10]. More recently, Ouyang et al. introduced a novel drill-bit arrangement method for CNC (Computer Numerical Control) woodworking machines under mass customization, achieving significant reductions in drilling time through clustering and intelligent optimization [11]. While these approaches contribute to equipment-level enhancements, they do not address information-layer machining logic or integration with MES (Manufacturing Execution Systems), both of which are essential for large-scale customized production.

With the growing adoption of cyber–physical production systems and MES-driven adaptive scheduling in the woodworking industry, the lack of flexible, information-layer drilling logic has become an increasingly critical bottleneck [12]. Given this gap, drilling process automation remains challenging in large-scale customized production environments, where frequent product switching and high variability in part design are common [13]. Existing solutions often depend on fixed machining logic or require manual reconfiguration, leading to increased equipment idle time, redundant slotting, and unprocessable horizontal holes near reference edges [14].

To address these limitations, the present study introduces an information-layer machining logic transformation that dynamically reconfigures drilling instructions before CNC execution. Unlike previous approaches focused on equipment upgrades or full-process redesign, this method aligns with cyber-physical manufacturing architectures, where decision rules are decoupled from physical equipment and embedded within the information layer. This strategy enables legacy CNC machines to perform tasks typically requiring dual-sided units or auxiliary milling modules, representing a novel contribution to intelligent woodworking production.

Building on this concept, this technical note proposes three targeted rule-based optimization strategies that address the major bottlenecks in current drilling workflows: (1) horizontal holes on reference edges that cannot be processed by single-sided drilling machines, (2) inefficient slotting operations traditionally performed via milling, and (3) excessive idle time caused by redundant flipping and dual-pass operations. Unlike prior approaches that focus on hard-ware modifications or full-process redesign, the proposed method introduces rule-based transformations that dynamically adjust machining logic before CNC execution. This strategy allows seamless integration with existing CNC equipment, edge banding units, and MES [15] supporting practical implementation without the need for physical system changes.

## Methods and experimental

To address the aforementioned challenges in the drilling process – namely, the inaccessibility of horizontal holes along fixed reference edges, low slotting efficiency, and pro-longed idle time of equipment – a set of optimization strategies was proposed, involving: (1) flipping panels along reference edges to relocate unprocessable holes, (2) extending slot length to enable sawing-based slotting, and (3) transforming hole-slot assignment logic to reduce redundant machining. These methods are designed to improve both the efficiency and accuracy of drilling operations in panel furniture manufacturing.

### Optimization of horizontal holes along reference edges

**Fig 1:**
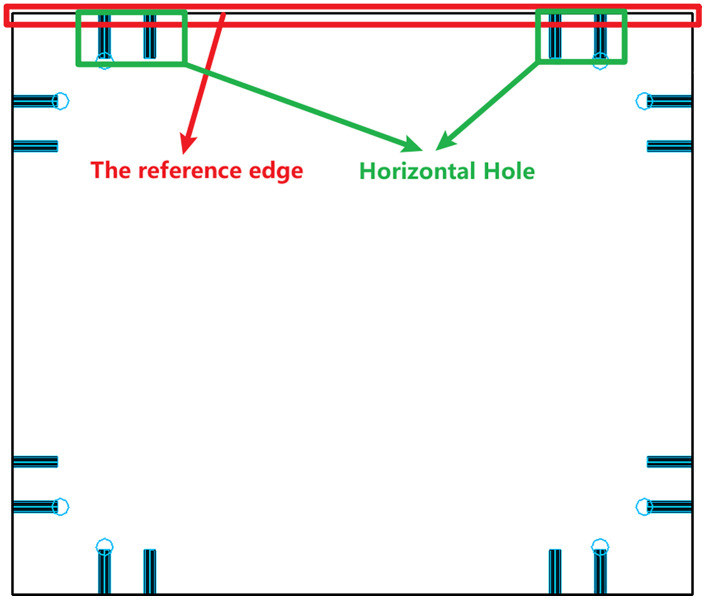
Original configuration: horizontal holes on the reference edge cannot be processed.

As shown in Fig 1, horizontal holes located on the fixed reference edge cannot be processed directly due to the mechanical limitations of certain CNC drilling machines. To overcome this, the proposed method introduces a flipping-based strategy. Specifically, horizontal holes that would otherwise be unprocessable are relocated to the reverse side of the panel drawing after a controlled flipping operation. The flipping is oriented along the horizontal axis of the reference edge, and the component is machined twice to ensure all holes are processed correctly.

**Fig 2:**
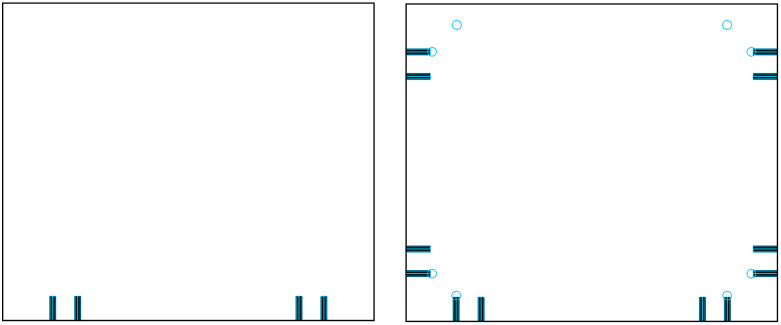
Optimized configuration: horizontal holes along the reference edge are reassigned to the reverse drawing. (a) Front-side drawing after optimization. (b) Reverse-side drawing showing reassigned hole positions.

The optimization procedure consists of the following steps:

a Analyze the drawing data to determine whether the panel has horizontal holes located along the reference edge;
b If such holes exist (as illustrated in Fig 1, temporarily store the hole information and modify the flipping strategy to orient the part along the horizontal direction of the reference edge;
c Check whether a reverse-side drawing already exists for the panel: (1) If a reverse drawing exists, the hole positions are recalculated based on the flipped coordinate system, and the stored hole data is reassigned accordingly. (2) If no reverse drawing is available, a new one is generated along with a corresponding drawing code. The stored hole data is then applied to the correct location on this new reverse drawing, as shown in Fig 2.

This flipping-based strategy enables accurate processing of horizontal holes that would otherwise be restricted by equipment limitations. By dynamically adjusting the flipping logic and drawing layers, the method maintains compatibility with existing production workflows and eliminates the need for additional hardware.

### Slotting efficiency enhancement via sawing transformation

**Fig 3:**
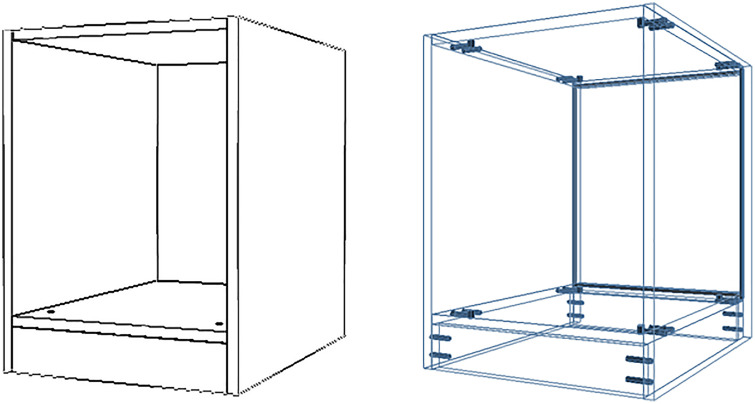
Cabinet design featuring grooved back-panel joints and an 80 ~ mm toe-kick. (a) Cabinet appearance rendering. (b) Structural diagram showing grooved panel configuration.

Panel furniture cabinets commonly employ grooved joints to embed back panels, which improves structural stability while reducing material usage [16]. Additionally, toe-kicks are typically installed at the base of the cabinet to protect against moisture and to facilitate door clearance [17]. Fig 3 shows an example of a cabinet structure with a toe-kick height of 80 mm and a grooved-panel design.

**Fig 4:**
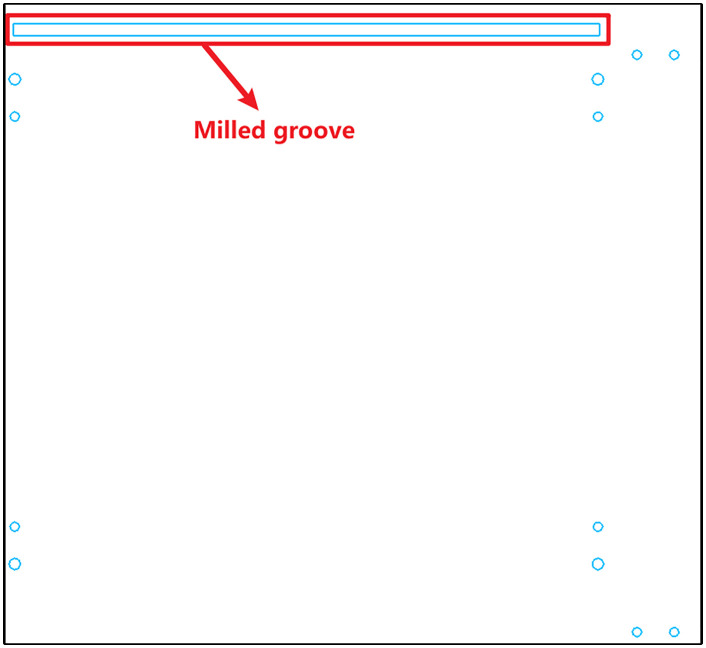
Original panel drawing showing milled groove for back panel insertion.

The drawing of the left side panel is shown in Fig 4, where the groove is machined by milling and serves as the insertion slot for the back panel.

In actual production, milling-based grooves are considerably less efficient than sawing-based grooves. If sawing can be used instead, it significantly improves processing speed. Moreover, edge banding machines in modern production lines can simultaneously perform sawing during the banding process. Specifically, when a panel requires a stand-ard groove positioned at a fixed distance (e.g., 18 mm) from the rear edge, the machine can automatically trigger a sawing operation by adjusting the blade vertically during edge banding [18].

**Fig 5:**
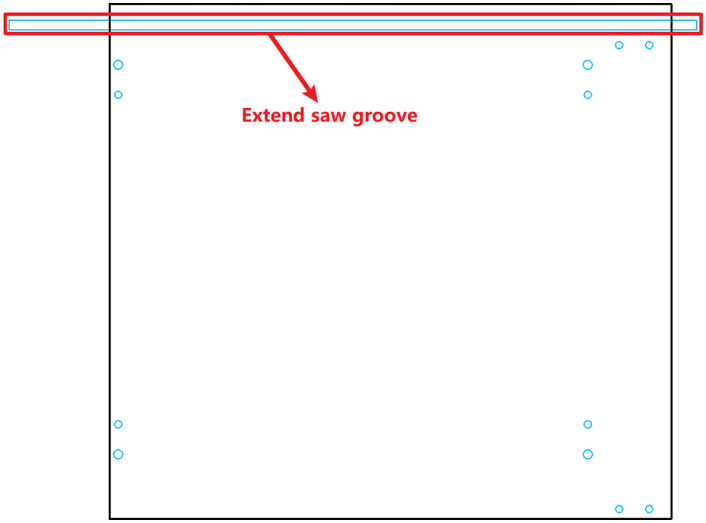
Converted groove structure: sawed groove with extended length beyond panel edges.

Based on this principle, the proposed method replaces milled grooves with sawed grooves and extends the groove length beyond the length of the panel, as shown in Fig 5. This allows standard slots to be processed during edge banding, thereby eliminating the need for separate slotting operations during the drilling stage.

The implementation consists of the following steps:

a Analyze the drawing information of cabinet panels and determine whether the current slotting method is milling;
b Identify the maximum toe-kick height as the basis for determining the extended slot length. Add an overflow value to both ends of the groove to ensure it exceeds the panel length;
c Transform the slotting method from milling to sawing, using the calculated extended length.

Although this method may result in grooves that extend beyond the panel edge, these extended portions are non-visible during assembly and do not affect aesthetics or functionality. By replacing milling with sawing, the slotting efficiency is significantly im-proved during the drilling stage.

Additionally, for panels with standard groove locations, the entire slotting operation can be preemptively handled by the edge banding machine, further streamlining the pro-duction workflow. This technique is particularly effective for improving the groove machining efficiency of cabinet top, bottom, and side panels in large-scale panel furniture production.

### Reduction of equipment idle time through hole-slot information transformation

As discussed in the previous section, when a panel contains a standard groove, the groove can be machined during the edge banding process, thus improving slotting efficiency. In such cases, the groove feature must be suppressed in the subsequent drilling process to avoid redundant machining.

However, in scenarios where a panel requires only a standard groove on the front side and additional drilling operations on the reverse side, single-sided CNC machines often encounter inefficiencies [19]. Specifically, the panel must first pass through the ma-chine without any processing, then be flipped and reloaded for reverse-side drilling [20]. This workflow leads to extended equipment idle time and disrupts the overall takt-time balance.

To address this issue, we propose a method for transforming the hole-slot assignment logic of such panels. The implementation procedure is as follows:

a Analyze the hole and groove information of each panel to identify those with: (1) standard grooves on the front side, and (2) required drilling operations on the reverse side. The groove must be confirmed as standard and feasible for edge banding machine processing.
b For panels meeting these criteria, transform the assignment so that the front side only contains drill holes, while the reverse side contains only the groove;
c During production, once the reverse-side standard groove has been completed by the edge banding machine, this status is communicated to the drilling stage via the MES. The drilling instructions are then updated accordingly, allowing the part to be machined in a single pass without requiring an additional flipping operation.

**Fig 6:**
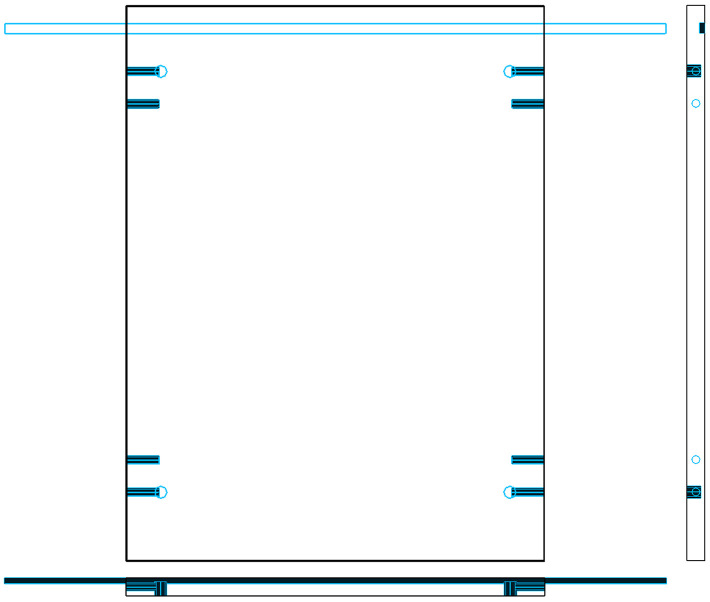
Three-view diagram of panel before hole-slot transformation. The front side contains a standard groove; the reverse side requires additional drilling.

**Fig 7:**
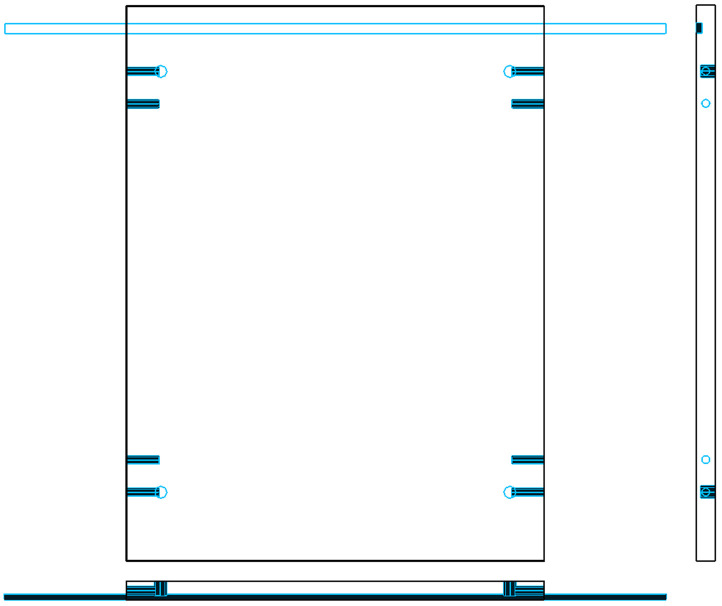
Modified three-view diagram after transformation. The groove is reassigned to the reverse side; the front side contains drill holes only.

Fig 6 shows the three-view drawing of the panel before transformation, while Fig 7 illustrates the modified version after hole-slot reassignment. It is important to note that this method is applicable only when the front and reverse sides of the panel use identical decorative surfaces, ensuring consistency in visual appearance after processing.

This strategy effectively reduces idle time in the drilling stage and contributes to smoother, more efficient production flows in panel furniture manufacturing.

### Integration options for the proposed logic-based methods

The three rule-based optimization strategies proposed in this study operate entirely at the information layer and focus on transforming drilling instructions before CNC execution. These methods can be deployed in two ways depending on the digital infrastructure of the enterprise.

For enterprises without a Manufacturing Execution System (MES), the logic transformations can be executed as a standalone pre-processing module that directly modifies the machining files (e.g., BPP, MPR, or G-code) generated from design drawings. This approach enables small and medium-sized manufacturers to benefit from the proposed strategies without requiring additional software systems.

For enterprises equipped with MES platforms, the methods can be embedded directly into the MES rule-processing layer. In this configuration, the logic transformations are applied automatically during the MES-to-CNC data transmission process, ensuring seamless integration with existing production workflows. The subsequent case study of Company W illustrates this MES-integrated implementation.

Because the proposed information-layer method does not alter spindle speeds, feed rates, tool geometry, or other machining conditions, drill wear and tool life remain unaffected and were not evaluated in this study. All drilling operations were carried out using Company W’s standard CNC tooling configuration and default machining parameters.

The proposed logic-based methods were tested on laminated particleboard and MDF panels and are applicable to other flat wood-based panels with similar geometric characteristics. In terms of applicability, the groove transformation and hole–slot reassignment strategies apply only to panels with (1) symmetric decorative surfaces on both sides, (2) standard back-panel groove positions, and (3) rectangular geometry. Panels with asymmetric finishes, special laminates, or curved or irregular profiles are excluded to prevent visual inconsistencies and ensure stability of machining results. For reproducibility, a unified pseudocode summarizing the three rule-based optimization strategies (flipping logic, groove transformation, and hole–slot reassignment) is provided in the S1 File.

## Results and discussion

**Fig 8:**
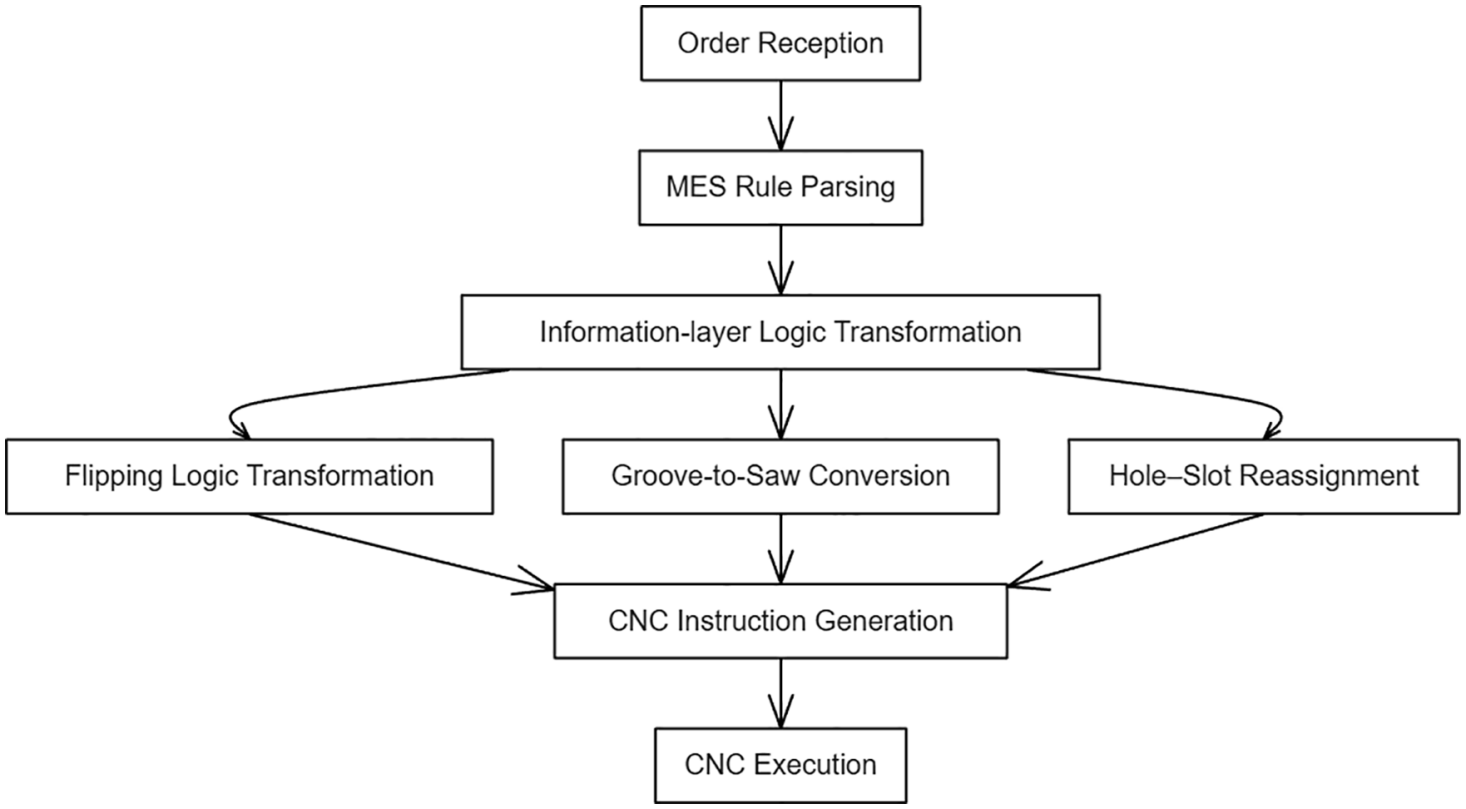
MES-integrated processing for optimized CNC machining instructions.

The proposed drilling process optimization methods were deployed in a production environment at a large-scale customized panel furniture manufacturer (referred to as Company W). During MES-to-CNC data transmission, the system automatically applies the flipping logic, groove transformation, and hole–slot reassignment rules, enabling the optimized machining instructions to be generated seamlessly before CNC execution. A simplified workflow of this MES-integrated processing is shown in Fig 8. The effectiveness of the three core strategies was verified individually using production data and analysis.

### Flipping strategy for horizontal hole machining

By implementing the flipping-based approach to reposition horizontal holes located on fixed reference edges, Company W successfully resolved the previous issue of unprocessable hole positions. Prior to this optimization, such panels were exclusively processed using the HOMAG PTP160 machine. Although technically capable, this equipment re-quired manual assistance and exhibited lower efficiency.

After the optimization was applied, single-sided CNC drilling equipment could be used instead, eliminating the need for manual intervention and improving production throughput. Historical production records from October to December 2024 indicated that, on average, 1331 panels per working day were redirected to the automated line using this strategy, significantly reducing the reliance on manual operations (see Table 1).

**Table 1:** Optimization summary and average daily processing volume at Company W.

Optimization Strategy	Total Panels	Workdays	Optimized Panels	Daily Avg. (%)
Flipping Strategy	2,957,442	74	98,482	1,331 (3.33%)
Slot Extension Strategy	2,957,442	74	1,250,966	16,905 (42.30%)
Hole-Slot Transformation	2,957,442	74	61,509	831 (2.08%)

### Slot extension and sawing-based grooving

Following the adoption of the slotting efficiency improvement method, Company W converted all panels with standard back grooves from milling to sawing. Additionally, rectangular panels with standard groove geometry were all processed by the edge banding machine during the banding stage, rather than during drilling.

This modification not only improved the groove machining speed but also prevented redundant machining by suppressing the back groove features in the drilling instructions. Over the three-month testing period, 42.30% of all panels were optimized using this method, resulting in an estimated 12% increase in production efficiency (see Table 1).

### Hole-slot logic transformation for equipment utilization

Before adopting the hole-slot transformation logic, panels with front-side grooves and reverse-side holes accounted for approximately 2.08% of the total production. These panels required two separate machining passes, with manual flipping between operations, leading to inefficient equipment usage (see Table 1).

After applying the proposed logic and embedding it into the company’s MES system, all qualified panels were automatically transformed for one-pass drilling [21]. The system now ensures that future eligible parts will be handled in the same optimized manner without additional configuration.

To further evaluate the efficiency improvement brought by the proposed hole-slot transformation method, a time-study experiment was conducted on 20 groups of panels. Prior to optimization, these panels required two separate drilling passes due to the presence of front-side grooves and reverse-side holes. After optimization, the panels were processed in a single pass.

A stopwatch-based continuous timing method was used to observe each processing unit. The drilling workflow was decomposed into six clearly defined operational units, as shown in Table 2. If a panel required drilling only on the front side, the process ended after Unit 3.

**Table 2:** Decomposition of drilling operation into six process units.

Unit No.	Total Panels
1	Panel enters drilling machine
2	Drilling front-side holes and grooves
3	Transfer to buffer platform or rotation station
4	Panel flipping time
5	Drilling reverse-side holes and grooves
6	Transfer to output platform

Twenty observations were recorded for each process unit, both before and after optimization. The data were analyzed using the three-sigma rule to detect and eliminate outliers. For each unit, the mean X̅, standard deviation σ, upper control limit (UCL = σ + 3*X̅), and lower control limit (LCL = σ – 3*X̅)) were calculated. No outliers were found in either dataset [22].

Table 3 and Table 4 summarize the actual operating times before and after optimization.

**Table 3:** Observed operation times before optimization (in seconds).

Obs.	Unit 1	Unit 2	Unit 3	Unit 4	Unit 5	Unit 6
1	2.742	3.855	5.305	33.731	29.472	5.497
2	2.879	3.515	5.751	38.303	18.886	5.644
3	2.337	3.938	5.088	34.853	31.455	5.494
4	2.958	3.349	5.521	37.926	11.253	5.655
5	2.875	3.379	5.260	33.782	37.280	5.732
6	2.701	3.933	5.824	39.337	14.215	5.271
7	2.439	3.975	5.663	32.290	26.720	5.750
8	2.733	3.979	5.933	30.275	23.720	5.203
9	2.665	3.114	5.724	30.605	29.068	5.407
10	2.428	3.282	5.113	33.915	39.177	5.749
11	2.693	3.079	5.197	39.366	15.994	5.330
12	2.827	3.707	5.945	36.094	14.286	5.426
13	2.417	3.867	5.290	30.905	37.114	5.702
14	2.572	3.000	5.746	36.448	34.914	5.165
15	2.304	3.269	5.087	31.932	10.138	5.100
16	2.621	3.691	5.025	33.447	13.985	5.482
17	2.008	3.702	5.520	31.152	14.638	5.560
18	2.387	3.746	5.947	33.559	22.983	5.779
19	2.858	3.658	5.848	33.240	13.883	5.010
20	2.878	3.754	5.239	33.999	34.076	5.516
X̅	2.616	3.590	5.501	34.258	23.663	5.474
σ	0.249	0.318	0.327	2.833	9.846	0.233
UCL	3.362	4.543	6.481	42.757	53.202	6.171
LCL	1.870	2.636	4.521	25.759	−5.876	4.776
Outliers	None	None	None	None	None	None

**Table 4:** Observed operation times after optimization (in seconds).

Obs.	Unit 1	Unit 2	Unit 3
1	2.720	20.715	5.691
2	2.980	33.062	5.496
3	2.718	33.233	5.576
4	2.166	12.571	5.857
5	2.337	22.411	5.748
6	2.734	20.639	5.089
7	2.429	36.840	5.646
8	2.651	33.756	5.007
9	2.996	15.706	5.922
10	2.742	10.230	5.931
11	2.337	22.362	5.504
12	2.757	27.807	5.584
13	2.386	14.382	5.040
14	2.605	31.250	5.513
15	2.095	24.417	5.468
16	2.830	24.462	5.644
17	2.233	27.955	5.186
18	2.330	25.576	5.572
19	2.273	37.597	5.297
20	2.329	25.008	5.294
X̅	2.532	24.999	5.503
σ	0.272	7.907	0.277
UCL	3.347	48.719	6.334
LCL	1.718	1.279	4.673
Outliers	None	None	None

The comparison of the results shows that the major time savings occurred during Unit 4 (panel flipping) and Unit 2 (front-side operation). The average total processing time per panel before optimization was 75.102 seconds, while after optimization, it was reduced to 33.035 seconds—resulting in a 56.01% improvement in production efficiency for these specific panel types.

## Discussion

The implementation of the proposed methods led to notable improvements in the drilling efficiency of panel furniture production at Company W. Each strategy addressed a specific bottleneck within the existing workflow:

- The flipping strategy enabled panels with horizontal holes on fixed reference edges to be processed automatically without specialized dual-sided machines;
- The conversion of milled grooves to sawed grooves, with extended lengths, allowed edge banding machines to perform slotting concurrently, reducing both tooling time and equipment switching;
- The reassignment of hole-slot logic reduced unnecessary panel flipping, idle ma-chine time, and operator involvement, particularly for panels with standard back grooves and reverse drilling features.

Quantitative evaluations confirmed the practical value of the methods. For example, 42.30% of the panels were optimized using the slotting transformation strategy, contributing to an estimated 12% increase in production efficiency. The hole-slot logic transformation enabled 2.08% of previously dual-pass panels to be machined in a single pass, improving takt-time and reducing labor demands. Time-study experiments showed a 56.01% improvement in processing speed for these panels, highlighting the effectiveness of the transformation in real industrial settings.

These findings align with current trends in smart manufacturing, which emphasize modular data-driven optimization within existing production constraints. Compared with previous studies focusing on physical redesign or hardware upgrades, the presented approach highlights the impact of information-level process reconfiguration.

From a broader perspective, the successful integration of the proposed logic into the MES environment demonstrates the feasibility of software-based adaptations in furniture manufacturing. It opens up opportunities for more advanced adaptive control systems that dynamically modify machining instructions based on part features, machine work-load, and real-time line balancing.

Detailed econometric or cost-benefit modelling of drilling operations (e.g., tool life, unit cost per hole) was not performed, as this study focuses on information-layer logic transformation rather than detailed economic analysis [23]. Because the reported improvements are derived from full production-line data rather than repeated laboratory trials, formal statistical inference (e.g., variance or confidence intervals) was not performed in this study.Future research could focus on adapting these optimization strategies to handle irregular or curved panel geometries, which are increasingly common in high-end custom furniture. Additionally, incorporating machine learning techniques may enhance the system’s adaptability by enabling real-time rule optimization and automatic parameter adjustment based on accumulated production data and performance trends [24].

## Conclusion

This study proposed an information-layer optimization framework to enhance drilling efficiency in mass-customized panel furniture manufacturing. By addressing key inefficiencies through flipping logic, groove transformation, and MES-integrated hole–slot reassignment, the methods demonstrated scalable, cost-effective, and equipment-compatible improvements to drilling workflows. The successful deployment in an industrial environment confirms the practical value and applicability of information-level process reconfiguration in modern manufacturing systems.

The proposed strategies apply primarily to panels with rectangular geometry, symmetric decorative surfaces, and standard back-panel groove positions. They have not yet been validated for irregular or curved shapes, multi-material laminated boards, or scenarios involving dynamic toolpath optimization under varying machine loads. Additionally, recommendations regarding drill design or surface-treatment optimization fall outside the scope of this study, as no tool-material or coating variables were modified.

Future research will focus on extending the applicability of these rule-based transformations to complex and non-rectangular geometries, as well as integrating real-time adaptive decision models to better respond to production variability. Machine-learning-based rule generation also represents a promising direction, enabling automated optimization of flipping, groove-processing, and hole–slot reassignment logic based on large-scale production data.

## Supporting information

S1 File Unified pseudocode for the proposed information-layer optimization strategies. (TXT)
